# COVID-19 impact on blood donation and blood product use in Mangaung Metropolitan Municipality

**DOI:** 10.4102/safp.v67i1.6177

**Published:** 2025-09-23

**Authors:** Karishma Naicker, Divya Ranchod, Kenny Msiza, Itumeleng Mosebetsane, Motshidisi Thulo, Nkosazana Mabunda, Sbongile Nyembe, Thandiswa Mpambani, Claire L. Barrett

**Affiliations:** 1 School of Clinical Medicine, Faculty of Health Sciences, University of the Free State, Bloemfontein, South Africa

**Keywords:** blood supply, blood donation, COVID-19, blood transfusion, health care

## Abstract

**Background:**

Blood donation rates in sub-Saharan Africa are historically low. The coronavirus disease 2019 (COVID-19) pandemic amplified this challenge, leading to significant declines in blood donations.

**Objective:**

This study aimed to assess the impact of COVID-19 on blood donation and utilisation in the Mangaung Metropolitan Municipality (MMM), South Africa, between April 2018 and March 2022.

**Methods:**

A retrospective analytical study was conducted using data from the South African National Blood Service (SANBS) Business Intelligence System. Blood donations and product utilisation were analysed across different time periods, aligning with COVID-19-related restrictions.

**Results:**

A substantial decrease in blood donations was observed during the pandemic. Blood collection decreased over all age groups but was particularly notable among younger donors (aged 15–39). Conversely, blood product utilisation increased across both public and private health care sectors during the pandemic.

**Conclusion:**

The COVID-19 pandemic had a profound impact on blood donation and utilisation in the MMM. To address future blood shortages, strategies are needed to encourage blood donation, optimise blood product utilisation and ensure equitable access to blood services.

**Contribution:**

The study contributes to understanding the impact of COVID-19 on blood donation and utilisation patterns in sub-Saharan Africa, specifically in the MMM in South Africa.

## Introduction

Blood shortages are a persistent challenge in sub-Saharan Africa (SSA), and the coronavirus disease 2019 (COVID-19) pandemic further exacerbated this issue.^1^ The early COVID-19 pandemic significantly impacted blood donation and also the clinical use of blood in Africa,^2^ with most countries seeing a reduction in both blood donation and clinical use of blood. Interestingly, the reduction in blood donations in South Africa was not as pronounced as in the rest of Africa (proportion decrease: −0.4 compared to −16.7 in South Africa and Africa, respectively). In South Africa, blood utilisation dropped by approximately 17% in the early pandemic, with a more pronounced initial decline in the public sector.^3^ Between April 2020 and March 2022, lockdown restrictions were enforced at varying levels in South Africa.

The South African National Blood Service (SANBS) relies on voluntary blood donations to meet the country’s blood needs, but less than 1% of the population actively donates blood, occasionally leading to shortages.^4,5^ Studies conducted in other regions, such as Europe, have shown that lockdown restrictions and fear of infection negatively impacted blood donation rates during the COVID-19 pandemic.^6^

Trauma and obstetric patients are especially reliant on blood products, which may be lifesaving.^1,7,8^ A study conducted from March 2020 to December 2020 reported a 22.7% increase in maternal deaths, with a 4.8% increase in neonatal deaths in South Africa. This study included a large sample of patients across multiple health care centres and found that the increase was statistically significant. The study did not specify whether this was all-cause mortality, but it did highlight the role of blood product shortage.^9^ The World Health Organization (WHO) reported that the shortage of blood products during the COVID-19 pandemic may have contributed to the increase in maternal and neonatal deaths and negatively affected the quality of maternal health.^8^ Local data from the Saving Mothers reports support this, with an increase in avoidable maternal deaths because of lack of blood products reported in the Eastern Cape (5.2% vs. 3.8% in 2020 compared to the 2017–2019 triennium). The Eastern Cape was the only province to show these data on a year-by-year basis in this report. It is concerning to note that the Free State had the highest percentage of maternal deaths because of avoidable factors attributed to lack of blood products (2.6%) in the 2020–2022 triennium. While maternal deaths increased annually from 2019 to 2021, with a return to baseline in 2022, the proportion of deaths related to obstetric haemorrhage remained stable.^10^

This study aimed to investigate the impact of COVID-19 on blood donation and utilisation patterns in the Mangaung Metropolitan Municipality (MMM), in the Free State, South Africa, between April 2018 and March 2022. By examining the data, we sought to understand the extent to which the pandemic affected blood supply and demand in this region.

## Research methods and design

### Study design

A retrospective analytical study was performed using data from the SANBS Business Intelligence System to assess blood donation and utilisation trends between April 2018 and March 2022. The system captures comprehensive data on blood donations, including donor demographics (age, sex), donor type (new, repeat, etc.) and the type of blood product collected. It also tracks clinical utilisation data, such as the number of units issued, recipient demographics and the hospital department where the product was used. The data are considered highly complete, as they are used for operational and management purposes by the SANBS. These time frames were chosen to represent the ‘pre-COVID period’ (April 2018–March 2020) and the ‘COVID period’ (April 2021–March 2022).^11,12^

South African National Blood Service aims to expand their donor pool by fostering understanding and building trust and educating potential blood donors using the media, social media and blood donor drives in communities and schools.^13^ Blood is collected at key population concentration points, using both mobile and fixed donor centres.^14^ In line with the WHO recommendation, all blood in South Africa is collected from voluntary, non-remunerated blood donors.^15^

Blood is used both in the public and private sectors. According to national trends, approximately 60.7% of blood is used in the public sector.^16^ Data on the provincial trends of public-to-private blood utilisation could not be found.

### Setting

The study was conducted in the MMM in South Africa’s Free State Province. The metro is home to a significant young working-age population (25–44-years-old, 31.8%) and young children (0–14-years-old, 25.6%).^17^ Blood products were prescribed and administered at both public and private health care facilities in this metro. Donors are primarily recruited through voluntary blood drives at schools, universities, workplaces and community centres, in addition to fixed SANBS donation sites.

### Data collection

Data on blood donation and clinical utilisation were extracted from the SANBS Business Intelligence System by SANBS collaborators. The data collected included donor demographics (age, sex), donor type (e.g. New, Repeat) and the number of units collected. For clinical utilisation, the data included the number of units issued, recipient demographics (age, sex), health care sector (public or private) and the hospital department. The data are comprehensive and are used for operational and management purposes by the SANBS, providing a high degree of confidence that all donations and prescriptions within the system were captured. South African National Blood Service provided the data in a Microsoft Excel spreadsheet. Data from 01 April 2018 to 31 March 2020 were defined as the pre-COVID-19 period, representing two full years of data prior to the start of the national lockdown in South Africa. The period from 01 April 2020 to 31 March 2022 was defined as the COVID-19 period, covering the two years of the pandemic and associated lockdown restrictions. The start date of the COVID-19 period, April 2020, was chosen to align with the implementation of the first national lockdown in South Africa.

The SANBS categorises their donors into five categories: New donors are those who have donated for the first time, join donors are those who have signed up to donate but have not yet donated, repeat donors are those who have made more than one donation, lapsed donors are those who have previously donated but have not donated for longer than 12 months and rejoin donors are lapsed donors who have started to donate again. The category of ‘Join Donors’ is included in this study to track the number of individuals presenting to donate and the potential donor base, even if they were not successful in their first attempt. While they did not contribute to the units of blood collected, their number provides insight into the potential donor pool and the impact of the pandemic on recruitment efforts.

### Data analysis

Data were analysed using descriptive statistics to compare trends before and during the COVID-19 pandemic.

The data analysis was conducted by the Department of Biostatistics at the University of the Free State (UFS). Data analysis was performed using R version 4.2.2 (R Foundation for Statistical Computing, Vienna, Austria). Categorical variables were summarised using frequencies and percentages, while numerical variables were summarised using means and standard deviations for normally distributed data and medians with interquartile ranges for skewed data.

To analyse trends in quarterly aggregated count data, Poisson regression was employed. The regression slopes were compared before and during the COVID-19 pandemic using a two-sample *t*-test with a pooled standard deviation. Statistical significance was set at a *p*-value of less than 0.05.

### Ethical considerations

The study protocol was reviewed and approved by the Health Sciences Research Ethics Committee of the University of the Free State (UFS-HSD2022/0430/3008) on 16 August 2022. Additionally, permission was obtained from the SANBS to access and analyse the necessary data from their database. South African National Blood Service REC also approved this study.

## Results

During the study period, 114 666 donations were made, yielding 95 442 units of blood. The number of units of blood (95 442) is less than the number of donations (114 666). A single donation may not always result in a usable unit of blood, for reasons such as a low haemoglobin count, or it may not have been fully processed. The term ‘units of blood’ refers to the final red cell concentrate available for transfusion (Table 1a). Concurrently, 96 083 units of red cell concentrate were issued for transfusion (Table 1b). The data in this study focus primarily on red cell concentrate, as this represents the most frequently transfused blood product. Results were determined by comparing variables prior to and during the COVID-19 pandemic.

**TABLE 1a:** Donations and donor data for the period prior and during COVID-19.

Donors	Category	Prior COVID-19 (*N* = 46 930)		During COVID-19 (*N* = 48 512)		Total	
		*n*	%	*n*	%	*n*	%
Donor sex (*n* = 95 442^†^)	Female	21 589	46.0	22 754	49.9	44 343	46.5
	Male	25 341	54.0	25 758	53.1	51 099	53.5
Donor type (*n* = 114 666^‡^)	Join	3041	5.2	1672	3.0	4713	4.1
	New	6846	11.1	4869	8.7	11 325	9.8
	Rejoin	7144	12.2	6611	11.7	13 755	12.0
	Repeat	41 759	71.5	43 114	76.6	84 873	74.0
Donor age (*n* = 95 442^†^)	15–19 years	5073	10.8	3908	8.1	8981	9.4
	20–29 years	11 748	25.0	11 095	22.9	22 843	23.9
	30–39 years	10 829	23.1	11 356	23.4	22 185	23.2
	40–49 years	9341	19.9	10 074	20.8	19 415	20.3
	50–59 years	6639	14.1	7791	16.1	14 430	15.1
	60–69 years	3010	6.4	3091	8.0	6911	7.2
	70–79 years	282	0.6	380	0.8	662	0.7
	80–89 years	8	0.02	7	0.01	15	0.02

COVID-19, coronavirus disease 2019.

† , The total number of donors is 95 442. This number refers to the number of individuals who successfully donated blood, from whom the units of blood were collected. The total number of donations (*n* = 114 666) includes all donation attempts, including join donors and lapsed donors, not all of which resulted in a successful donation.

‡ , The sum of the donor type categories (join, new, rejoin and repeat) (*n* = 114 666) includes all donors who presented to donate, not just those who were successful in donating. The number of successful donations was 95 442, which is why the donor sex and donor age categories sum up to this number.

**TABLE 1b:** Recipient data for the period prior and during COVID-19.

Recipients (*n* = 96 083)	Category§	Prior COVID-19 (*N* = 45 883)		During COVID-19 (*N* = 50 200)		Total (*N* = 96 083)	
		*n*	%	*n*	%	*n*	%
Sex	Female	26 239	57.2	28 588	56.9	54 827	57.1
	Male	19 639	42.8	21 583	43.0	41 222	42.9
	Unknown	5	0.01	29	0.06	34	0.04
Health care sector	Private	25 303	55.1	28 200	56.2	53 503	55.7
	Public	20 580	44.9	22 000	43.8	42 580	44.3
Age (years)	> 1	1855	4.0	2489	5.0	4344	4.5
	1–9	2264	4.9	2676	5.3	4940	5.1
	10–19	1406	3.1	2264	4.5	3670	3.8
	20–29	4097	8.9	5026	10.0	9123	9.5
	30–39	6649	14.5	8048	16.0	14 697	15.3
	40–49	6159	13.4	6691	13.3	12 850	13.4
	50–59	6424	14.0	7305	14.6	13 729	14.3
	60–69	7254	15.8	7090	14.1	14 344	14.9
	70–79	6029	13.1	5804	11.6	11 833	12.3
	80–89	3048	6.6	2346	4.7	5394	5.6
	≤ 90	698	1.5	461	0.9	1159	1.2
Department	Cardiothoracic surgery	1235	2.7	1799	3.6	3034	3.2
	General surgery	4035	8.8	3994	8.0	8029	8.4
	Gynaecology and Obstetrics	5199	11.3	6609	13.2	11 808	12.3
	Haematology and Oncology	3800	8.3	3265	6.5	7065	7.4
	Intensive care unit (ICU)	10 958	23.9	9866	19.7	20 824	21.7
	Infectious complications	61	0.1	35	0.1	96	0.1
	Medical	11 339	24.7	15 024	29.9	26 363	27.4
	Orthopaedic	1724	3.8	1333	2.7	3057	3.2
	Paediatric surgery	170	0.4	131	0.3	301	0.3
	Paediatrics	1886	4.1	2034	4.1	3920	4.1
	Trauma	1383	3.0	1948	3.9	3331	3.5
	Other	4093	8.9	4162	8.3	8255	8.6

COVID-19, coronavirus disease 2019.

§ , These categories are as per the SANBS blood requisition form and are reported as such.

### Blood donor type and units of blood collected

A statistically significant decrease (*p* < 0.0001) was observed in all four blood donor categories during the COVID-19 pandemic compared to the pre-pandemic period. This comparison was made using Poisson regression to analyse the trends in quarterly aggregated count data. The dependent variable was the quarterly count of donations or units collected, and the predictor variable was the time quarter. The regression slopes were compared before and after the COVID-19 pandemic using a two-sample *t*-test with a pooled standard deviation. The coefficients and confidence intervals are available upon request. While new, rejoin and repeat donors maintained a positive trend in blood donations before the pandemic, the slope for join donors decreased from positive (0.355) to negative (−0.046) during the pandemic. This demonstrates that recruitment efforts for first-time donors were particularly impacted.

**FIGURE 1:**
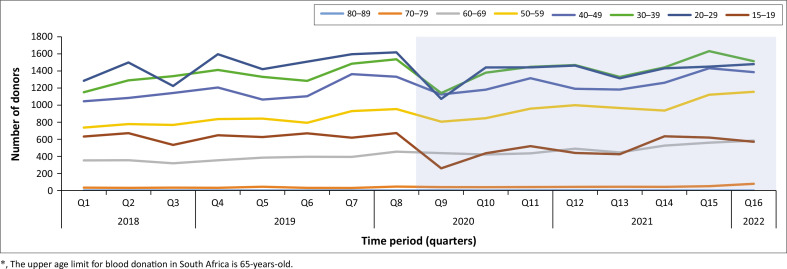
Blood units collected according to donor age prior to and during COVID-19.

Figure 1 demonstrates a decrease in blood donations across all age groups in Quarter 9 (2020). This decline was most pronounced among individuals aged 15–39 years. However, a decline was observed across all age groups although the impact was most severe in the younger demographics. The decrease was also transient, with donation numbers beginning to recover as lockdown restrictions were eased.

**FIGURE 2:**
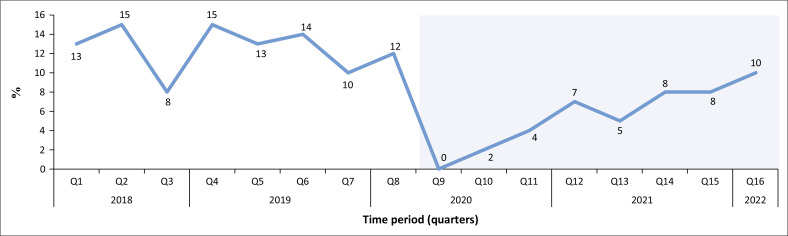
Percentage of student and young adult donors prior to and during COVID-19.

On average, student and young adult donors constituted 8% of donations in the study period. This group, defined as 15–39-years-old, contributed 56.5% of total donations, with the 20–29-years age group alone contributing 23.9%, as shown in Table 1a. In the pre-COVID period, students and young adults constituted 12.5% and 5.5% in the COVID period. The percentage of student and young adult blood donors decreased from 12% to 0% of total donors from Quarter 8 to Quarter 9 (2020) (Figure 2). The decrease in the percentage of student and young blood donors from 12% to 0% of total donors from Quarter 8 to Quarter 9 (2020) was statistically significant (*p* < 0.0001). Between Quarter 12 and Quarter 13 (2021), there was another decrease from 7% to 5% of total donors, which was within the normal range of fluctuation seen in the pre-COVID data.

Female donors were predominant during the COVID-19 pandemic because their slope was more positive (−0.061) compared to that of male donors (−0.106).

**FIGURE 3:**
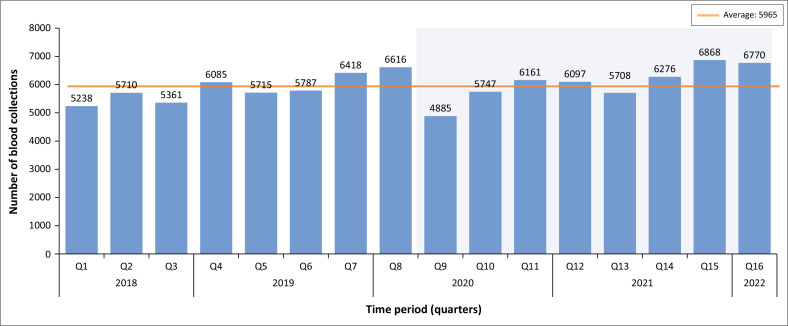
Units of blood collected prior to and during COVID-19.

Quarter 9 (2020) had the lowest number of blood collections, which was 4885 (Figure 3) and was below the average number of collections throughout the study period.

**FIGURE 4:**
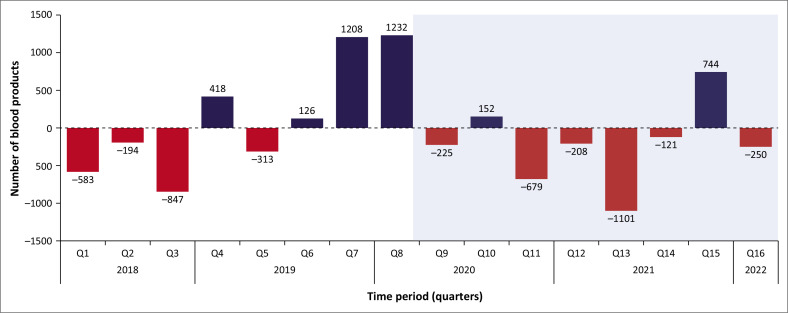
Blood products collected versus blood products billed in the Mangaung Metropolitan Municipality.

Before the COVID-19 pandemic, four out of eight quarters showed a deficit in the collected blood products compared to those billed in the MMM. During the COVID-19 pandemic, six out of eight quarters exhibited a deficit (Figure 4).

### Clinical use of blood products by hospital, department, health care sector and recipient demographic

**FIGURE 5:**
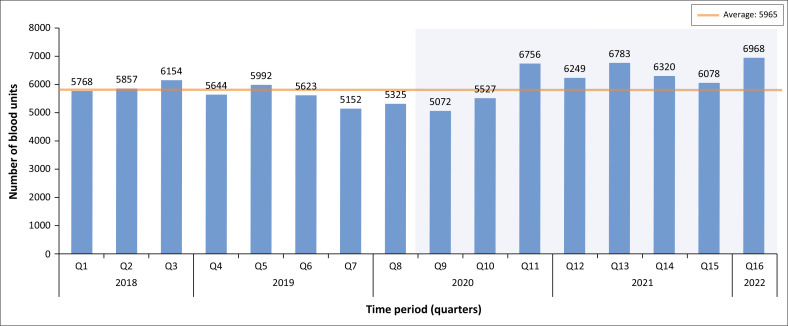
Blood billed to hospitals.

Throughout the study period, an average of 5954 units of blood were billed per quarter in the MMM. In Quarter 9 (2020), coinciding with the beginning of the pandemic, the number of blood products billed was the lowest (5072 units). Following the early stages of the pandemic, the usage of blood products increased considerably, which is clearly seen in Quarter 11 (2020), Quarter 13 (2021) and Quarter 16 (2022) (Figure 5).

The use of red cell concentrate (RBC) increased significantly from before the COVID-19 pandemic to during the COVID-19 pandemic (*p* < 0.0001). Apart from *infectious complications*, a statistically significant increase in the clinical use of blood products during the COVID-19 pandemic compared to the period before the COVID-19 pandemic (*p* < 0.0001) was noted in all therapeutic areas (Table 2).

**TABLE 2:** Usage of blood products by hospital department.

Department	Slope before COVID-19	Slope during COVID-19	*p*
Cardiothoracic surgery	−0.119	0.270	< 0.0001
General surgery	−0.256	0.196	< 0.0001
Gynaecology and Obstetrics	−0.047	0.243	< 0.0001
Haematology and Oncology	0.006	0.305	< 0.0001
Intensive care unit (ICU)	−0.159	0.280	< 0.0001
Infectious complications	1.315	0.315	0.0179
Medical	−0.077	0.442	< 0.0001
Orthopaedic	−0.162	0.255	< 0.0001
Paediatric surgery	−0.347	0.538	< 0.0001
Paediatrics	−0.081	0.413	< 0.0001
Trauma	−0.111	0.292	< 0.0001
Other	−0.163	0.393	< 0.0001

COVID-19, coronavirus disease 2019.

Regarding the usage of blood products billed in the public health care sector, there was a negative slope before the pandemic at −0.091, which changed to a positive slope during the pandemic (0.354). The private health care sector showed the same trend, being −0.066 prior to the pandemic and 0.333 during the pandemic. For both the private and public health care sectors, a statistically significant difference in the slopes was noted (*p* < 0.0001). Throughout the study period, the private health care sector used 55.7% of the blood products.

In the public health care sector, an increase in the use of blood products during the pandemic was noted for the following age groups: 0–9-years-old, 20–29-years-old, and 30–39-years-old. The number of blood products used for patients above 40-years-old decreased during the COVID-19 pandemic. In the private health care sector, there was an increase in the use of blood products in all age categories between 0–69-years-old, whereas there was a decrease in the usage of blood products for individuals 70-years-old and older.

Before the COVID-19 pandemic, both male and female recipients had positive slopes, 0.358 and 0.329, respectively, indicating a slight increase in the number of blood products received over time. During the pandemic, they both changed to negative slopes with −0.061 for females and −0.106 for males (*p* < 0.0001), indicating a decrease in the number of blood products received. Throughout the study period, females received more blood products than men. If the Gynaecology and Obstetrics department is disregarded, the number of blood products received by males is higher than that of females.

**FIGURE 6:**
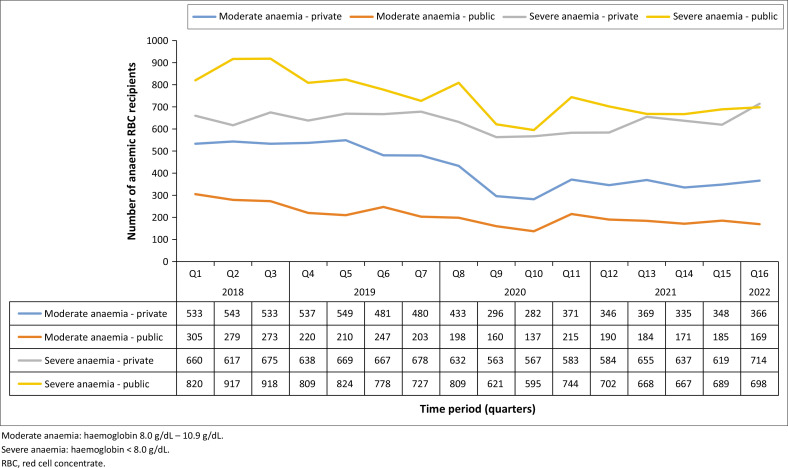
Severity of anaemia in red cell concentrate recipients according to health care sector.

Most recipients of RBC (Figure 6) were severely anaemic (haemoglobin < 8.0 g/dL) patients from the public sector, followed by severely anaemic patients in the private sector. Patients with moderate anaemia (haemoglobin 8.0 g/dL – 10.9 g/dL) in the public sector represented the smallest proportion of recipients.

## Discussion

This study investigated the effect of COVID-19 on blood donations and the clinical usage of blood products in the MMM. We describe a decrease in the number of blood donors in the MMM following the announcement of the lockdown in Quarter 8 (2020), with an increase in the amount of blood used in the same period.

The decrease in blood donations during the COVID-19 pandemic has previously been ascribed to the healthy population’s fear that they would contract COVID-19 in high-risk settings such as health care facilities.^1,18^ National lockdowns and quarantining made it difficult to move around normally, changes to the workplace disturbed regular routines, there was a lack of certainty regarding the availability of blood collection services, and blood drives were cancelled.^6^

Even under lockdown level 5 restrictions, the South African government permitted blood donors to donate blood as an essential service at fixed SANBS donation sites after undergoing rigorous contact tracing and screening procedures to ensure a sufficient blood supply.^5^ However, movement restrictions still discouraged regular and potential donors from travelling to the fixed donation sites. The closure of schools that were regularly used as mobile donation sites by SANBS also had an impact on blood donations in the MMM. This is demonstrated by the number of blood donations that declined across all age groups, with a decline noted among those who were scholars and young adults, 15–39-years-old. This age group consists mainly of the student population and young adults, representing a large group of donors in the MMM. When lockdown restrictions were strengthened from adjusted level 2 to adjusted level 4 in the second quarter of 2021,^19^ there was a decrease in blood donations made by students and young adults.^6^ As lockdown restrictions were eased, the total number of blood donations increased, surpassing the average.^4,6^

The SANBS typically aims for a blood supply of five to seven days. The observed deficits in both the pre- and post-COVID periods suggest that the region’s blood supply was often below this desired level, which is a persistent challenge for the SANBS. In the two years before the COVID-19 pandemic, there were periods where the demand for red cell concentrate exceeded the number of units collected, leading to occasional blood product shortages. Following the onset of the pandemic, these shortages became more frequent, with an increasing number of sectors experiencing deficits in blood supply.^5,7,8^ Shortages of blood products may result in cutbacks, where fewer units of blood are issued than requested. A lack of self-sufficiency of blood products in the anonymised, irrespective of the COVID-19 pandemic, is noted, with fewer units donated compared to units transfused.

A notable shift in transfusion patterns was observed at the beginning of the pandemic. In the eight quarters prior, fewer blood products were transfused, but this changed with an increase in blood product prescriptions. The reasons for this shift remain unclear. Initially, the postponement of elective admissions and surgical procedures would have been expected to reduce transfusion demand. However, patients with prevalent comorbidities – such as obesity, diabetes and hypertension – were more vulnerable to severe COVID-19,^20,21^ often requiring hospitalisation for complications like acute respiratory distress syndrome, acute kidney injury or multiorgan failure, which often lead to conditions such as anaemia and coagulation dysfunctions that necessitate blood transfusions.^21^ Additionally, increased transfusions were needed to manage anaemia and coagulation dysfunctions associated with COVID-19, further driving blood product utilisation.^3,22,23^ The rise in transfusions following the easing of lockdown restrictions may also be linked to efforts to address the surgical backlog created during the pandemic.^23^

Data from the Tygerberg’s surgery department (at Tygerberg Hospital in the Western Cape) showed a decrease in the use of blood products during the pandemic.^4^ In Harry Gwala Regional Hospital, a similar trend was noted with a decrease in the use of blood products during the lockdown, with an increase during the weekend straight after lifting the alcohol ban.^24^ Suggested reasons for the decreased use of blood products were the implementation of movement restrictions and the ban on alcohol. This is supported by a decrease in motor vehicle accidents, pedestrian vehicle accidents and interpersonal violence reported at Grey’s Hospital in KwaZulu-Natal during the COVID-19 lockdown period.^25^ Similarly, a study from Tshepong Hospital in the Northwest Province showed a 66% decrease in traumatic brain injuries in level 5 lockdown periods, with a ‘rebound trauma’ effect, with more traumatic brain injuries noted in level 2 and 3 lockdown.^26^ However, the data from the MMM in our study show an increase in the use of blood products in the trauma therapeutic area during the pandemic, which requires further investigation. We did not analyse the variations in the eight quarters in this therapeutic area, which would have added value. We anticipate that it may have fluctuated depending on the level of lockdown, like the data presented by Bulabula et al.^26^

Blood transfusion serves as a meaningful indicator of health care access in South Africa’s public and private sectors. As a vital medical intervention, its availability reflects the efficiency of blood supply systems, the effectiveness of donation programmes and the ability of health care facilities to provide timely treatment. In 2020, only 14.78% of South Africans were enrolled in a medical scheme, yet our study found that 55% of the country’s blood supply was utilised in the private sector. Research by Swanevelder and colleagues^3^ further highlighted sectoral disparities during the COVID-19 pandemic, showing that the public sector experienced a sharper decline in blood usage and a slower return to baseline levels compared to the private sector. Their national study revealed that blood usage in private facilities was 3 to 3.7 times higher than in public hospitals, underscoring potential differences in access to care during the pandemic. These disparities in transfusion practices may reflect broader inequities in health care access, as well as varied transfusion thresholds between clinicians and differing implementation of restrictive transfusion practices. By examining blood availability and utilisation patterns, researchers and policymakers can gain critical insights into systemic inequalities and identify strategies to enhance equitable health care delivery.

Findings from other studies support our assertion that the COVID-19 pandemic had a detrimental effect on both blood donations and transfusions.^24,27,28,29^ Consequently, this study contributes to the existing body of knowledge on blood donation patterns, particularly highlighting donor behaviour during crises – specifically, the reluctance to donate in such periods. Understanding these trends may help policymakers better prepare for future pandemics and assist clinicians in making informed decisions between transfusion and alternative treatments, such as haematinics for anaemic patients, to conserve blood products for those in critical need.^9,29^ Ensuring a stable blood supply during crises requires strengthening awareness around blood donation, which in turn necessitates dedicated government resources and strategic planning.

### Limitations of the study

The data were limited according to available clinical data from the SANBS Business Intelligence System. Some data from the Northern Cape may have been included in the donor data because of SANBS operational areas. Usage data are all for the MMM.

## Conclusion

This study showed that during the comparison periods of 01 April 2018 to 31 March 2020 (pre-COVID) and 01 April 2020 to 31 March 2022 (during COVID), the COVID-19 pandemic did affect the number of blood donations and the clinical use of blood products in the MMM.

### Recommendations

Further studies can investigate how to improve the number of blood donations so that the blood products collected and the blood products billed can be balanced in the future. Furthermore, investigations can be conducted regarding disparities in the use of blood products between the public and private health care sectors.
